# CP-LDS-MCTS: A Decision-Making Method for Unsignalized Intersections Based on Low-Discrepancy Sampling and Safety Pruning

**DOI:** 10.3390/s26092704

**Published:** 2026-04-27

**Authors:** Ning Sun, Jiahao Yu, Yantai Gao, Guangbing Xiao

**Affiliations:** College of Automobile and Traffic Engineering, Nanjing Forestry University, Nanjing 210037, China; ningsun@njfu.edu.com (N.S.); yujiahao_njfu@163.com (J.Y.); gyt1110@hotmail.com (Y.G.)

**Keywords:** autonomous driving, unsignalized intersections, Monte Carlo tree search, control barrier function, low-discrepancy sampling

## Abstract

Unsignalized intersections pose a representative challenge for autonomous-driving decision-making because online planning must satisfy tightly coupled requirements for safety, task completion, traffic efficiency, and control smoothness under a limited computation budget. Existing continuous-action MCTS planners often suffer from sparse candidate-action coverage and from the absence of an internal safety filter before node expansion. To address these issues, this paper proposes CP-LDS-MCTS, a decision-making framework that coordinates Sobol low-discrepancy sampling, truncated Taylor control barrier function (TTCBF)-based safety pruning, and policy-value composite scoring within the expansion stage of Monte Carlo tree search. Sobol sampling improves candidate representativeness under a fixed sampling budget; TTCBF provides a local one-step screening rule that removes actions inconsistent with safety constraints before search resources are consumed; and composite scoring prioritizes safe actions that are simultaneously policy-consistent and value-promising. To clarify the methodological contribution, CP-LDS-MCTS is formulated as a unified expansion-stage design rather than a loose combination of independent modules. The revised manuscript further adds a local approximation-error discussion for the TTCBF truncation, a computational-complexity analysis, a real-time latency evaluation, statistical significance tests, and two stronger baselines, namely PPO and MPC-CBF. Experiments in CARLA Town03 under low-, medium-, and high-density traffic show that the proposed method achieves the best overall balance among safety, success rate, travel time, and control smoothness while maintaining a mean planning latency below 25 ms per step on the test platform. The resulting safety assurance is local rather than global, as TTCBF pruning performs a one-step approximation-based feasibility check within the expansion stage and is validated in simulation. These results suggest that candidate coverage, internal safety screening, and value-aware expansion should be designed jointly for real-time continuous-action planning at unsignalized intersections.

## 1. Introduction

### 1.1. Research Background and Challenges

Unsignalized intersections are among the most challenging interactive scenarios in urban autonomous driving. Without explicit signal control, the ego vehicle must reason about uncertain right-of-way relations, anticipate the motion of surrounding vehicles, and generate safe and efficient actions within a short planning horizon. Compared with lane following or signalized intersection driving, this scenario imposes substantially higher demands on online planning because safety, task completion, and traffic efficiency are tightly coupled and must be balanced in real time.

In recent years, planning frameworks that combine neural guidance with tree search have shown strong potential for complex sequential decision-making problems. The AlphaGo family demonstrated how policy priors can guide exploration while value estimation reduces the reliance on expensive random rollouts, and related ideas have subsequently influenced robotics and autonomous driving [1,2]. For interactive driving scenarios, this line of research is particularly attractive because it preserves the structured search behavior of Monte Carlo tree search (MCTS) while introducing learned priors that improve search directionality under strict online computational budgets.

### 1.2. Current State of Research

Research on autonomous-driving decision-making has gradually evolved from rule-based strategies to learning-based planners and, more recently, to hybrid methods that combine learning, search, and safety constraints. Recent surveys have emphasized that effective decision systems must jointly handle interaction reasoning, safety assurance, and computational efficiency, especially in unsignalized intersections and mixed-traffic environments [3,4,5,6]. For the present problem—continuous-action search with internal safety screening—existing work can be organized into four main categories.

(1) Search expansion and MCTS planning in continuous action spaces. MCTS and its variants provide a general framework for sequential decision-making under online budget constraints [7]. In continuous spaces, progressive widening and related ideas are commonly used to control branching [8,9]. In autonomous driving, MCTS-based planners have also been applied to speed regulation, lane changing, and safety-enhanced decision-making [10,11]. However, most existing studies focus on how to search after candidate actions have already been produced, whereas the quality of the pre-expansion candidate set under a fixed budget remains comparatively underexplored.
(2) Learning-driven behavioral planning and interactive decision-making. Reinforcement learning, graph reasoning, and policy priors have been increasingly used to improve decision quality in interactive driving scenes [1,2,12]. Representative studies include constrained multi-objective reinforcement learning for personalized driving [13], predictive trajectory planning at intersections [14], and reasoning-graph reinforcement learning for mixed human–machine traffic at unsignalized intersections [15]. These approaches improve adaptability, but their effectiveness still depends strongly on the quality of the candidate-action set and on how safety constraints are handled during online planning.
(3) Safety-constraint-driven intersection planning and control. Responsibility-sensitive safety, optimal control, and control barrier functions provide formal tools for safety assurance in interactive traffic systems [16,17]. Recent CBF literature has further clarified practical issues such as high relative order, sample-and-hold implementation, and real-time feasibility [18,19,20]. In addition, adaptive CBF formulations have recently been explored for autonomous intersection management [21]. While these studies provide strong safety foundations, they are typically positioned at the control or optimization layer and are only loosely coupled with the candidate-expansion stage of continuous-action tree search.
(4) Mixed-traffic coordination and trajectory planning at intersections. Another research stream studies the coupled relationship between efficiency and safety from the perspectives of trajectory planning, cooperative control, and traffic organization [4,22,23,24,25]. These studies consistently indicate that high-density intersection performance depends on a coordinated balance among safety, completion rate, and efficiency rather than on isolated improvement of a single metric. Nevertheless, they do not explicitly resolve how candidate coverage, internal safety filtering, and long-horizon action ranking should be jointly organized inside a single online search pipeline.

In summary, existing research has made substantial progress in continuous search, learning-driven decision-making, safety-constrained control, and mixed-traffic planning. Yet three issues remain insufficiently resolved for online continuous-action planning at unsignalized intersections: (i) candidate actions may be poorly distributed under a limited sampling budget; (ii) many planners lack an explicit internal mechanism to reject locally unsafe actions before expansion; and (iii) the interaction among candidate coverage, safety filtering, and value-aware expansion priority is rarely treated as one unified design problem. The novelty of the present work lies in addressing this coupled expansion-stage problem directly, rather than improving only one isolated module.

### 1.3. This Work and Its Main Contributions

To address these limitations, this paper proposes CP-LDS-MCTS, a continuous-action planning method for unsignalized intersections. The central methodological idea is to cast node expansion as a budgeted allocation problem: under a fixed online computation budget, the planner should first generate representative candidate actions, then reject locally unsafe ones before expansion, and finally concentrate search on the safe actions with the highest strategic utility. CP-LDS-MCTS implements this idea as one coherent expansion-stage pipeline rather than as a loose juxtaposition of sampling, safety filtering, and action ranking modules.

(1) We formulate the expansion stage of continuous-action MCTS for unsignalized intersections in a unified manner, explicitly linking state representation, vehicle dynamics, local safety-set modeling, and policy-value-guided search within one online planning framework.
(2) We design a coordinated candidate-expansion mechanism. Sobol low-discrepancy sampling improves candidate coverage under a fixed budget, whereas TTCBF-based pruning screens out actions that violate local one-step safety conditions before search effort is spent on them. In the revised manuscript, we further clarify the approximation assumptions and interpretation boundary of this pruning rule.
(3) We introduce a policy-value composite scoring rule for elite-action selection and strengthen the empirical study with a complexity discussion, runtime analysis, stronger baselines (PPO and MPC-CBF), and statistical tests. Under the present CARLA evaluation setting, the resulting framework achieves a better overall balance among safety, task completion, efficiency, and smoothness than the compared baselines.

## 2. Problem Formulation and System Modeling

### 2.1. Scenario Description and Basic Assumptions

**Figure 1:**
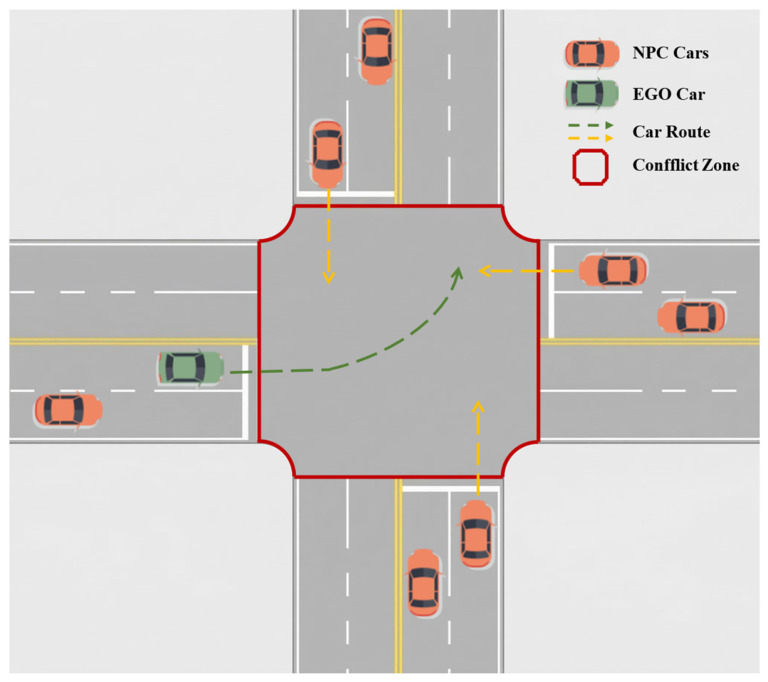
Schematic diagram of the four-way unsignalized intersection considered in this study.

This study considers the four-way unsignalized intersection shown in Figure 1. Without fixed signal control, the ego vehicle must decide whether to yield, follow, pass through, or brake based on its own state and the observed motion of nearby vehicles. The planning problem is therefore highly interactive and time-sensitive: the action chosen at the current step affects not only immediate collision risk but also the feasibility and quality of later decisions:

(1) The perception and tracking module can provide reliable observations of the ego vehicle and nearby traffic participants within the local planning region, and the observation error within one planning cycle is assumed to be bounded and comparatively small;
(2) The paper focuses on online planning for a single intelligent ego vehicle. Other traffic participants are modeled as dynamic elements of the environment rather than as explicitly coordinated decision-making agents, although their observed states are encoded in the local traffic representation;
(3) Because the planning horizon is short, the tree-search module uses first-order local motion extrapolation to predict background vehicles during single-step expansion. This approximation reduces computational overhead while retaining a usable estimate of short-horizon interaction risk;
(4) Within the intersection area, the ego vehicle typically operates at low to moderate speed; therefore, a kinematic bicycle model is used to describe its motion.

These assumptions do not imply that other traffic participants are treated as static or rigid obstacles. Rather, the planner continually uses real-time observations of nearby vehicles to form a local interaction state, and the resulting method should be understood as a computationally tractable online planner under short-horizon prediction assumptions rather than as a full multi-agent game-theoretic solution.

### 2.2. Vehicle Kinematic Model

Considering the low- to medium-speed operating regime typical of urban intersections, this paper uses a standard kinematic bicycle model to describe vehicle motion in the two-dimensional plane. Let the wheelbase be *L*, the rear-axle center position be $\left(x,y\right)$, the heading angle be ψ, the longitudinal speed be v, the longitudinal acceleration input be $a_{lon}$, and the steering angle input be $\delta_{f}$. The discrete-time state update is then written as follows:

(1) $$\left\{\begin{array}{c} x_{t+1}=x_{t}+v_{t}\cos\psi_{t}\cdot \Delta t\\ y_{t+1}=y_{t}+v_{t}\sin\psi_{t}\cdot \Delta t\\ \psi_{t+1}=\psi_{t}+\frac{v_{t}\tan\delta_{f}}{L}\cdot \Delta t\\ v_{t+1}=v_{t}+a\cdot \Delta t \end{array}\right.$$

Here, Δt represents the discrete time step. This model features a simple structure and high interpretability, making it suitable for embedding within online planning and tree search algorithms. Under the low-speed operating conditions of urban intersections considered in this paper, the model achieves a favorable balance between computational efficiency and modeling accuracy.

### 2.3. State Space and Action Space Definitions

To capture both ego dynamics and local interaction structure, the system state at time *t* is defined as follows:

(2) $$s_{t}=\left[\begin{array}{c} s_{t}^{ego},s_{t}^{nbr},s_{t}^{ctx} \end{array}\right]$$

where $s_{t}^{ego}=(x,y,\psi ,v,a_{lon})$ represents vehicle status, and the scalar acceleration component inside the state denotes the current longitudinal acceleration state component rather than the full control vector; $s_{t}^{nbr}$ represents local neighborhood traffic flow observations, comprising the $K_{nbr}$ most relevant vehicles within the planning scope, with each vehicle’s characteristics recorded as:

(3) $$s_{t}^{nbr,i}=(\Delta x_{i},\Delta y_{i},\Delta v_{x,i},\Delta v_{y,i},\Delta \psi_{i},d_{i}^{conf}),i=1,\ldots ,K$$

Variables $\left(\Delta x_{i},\Delta y_{i}\right)$ and $\left(\Delta v_{x,i},\Delta v_{y,i}\right)$ represent the relative position and relative velocity of the target vehicle relative to the self-vehicle, respectively. $\Delta \psi_{i}$ denotes the relative heading, while $d_{i}^{conf}$ indicates the distance from the target vehicle to the nearest potential conflict zone. $s_{t}^{ctx}=(d_{min},N_{conflict},\kappa )$ encompasses scene context features, including the distance to the nearest obstacle, the number of potential conflict vehicles, and the curvature of the road centerline. To maintain a fixed input dimension, zero padding is applied when the number of adjacent vehicles is less than $K_{nbr}$. All variables undergo standardized processing before being input into the neural network.

The action space is defined as a two-dimensional continuous control vector:

(4) $$u_{t}=(a_{lon},\delta_{f})$$

where $a_{lon}\in [a_{min},a_{max}]{m/s}^{2}$ denotes the longitudinal acceleration control input, and $\delta_{f}\in [\delta_{min},\delta_{max}]$ denotes the front wheel steering angle control input. This continuous two-dimensional action definition preserves the coupling between longitudinal and lateral control while remaining convenient for candidate generation, safety screening, and search expansion.

### 2.4. Definition of Safety Constraints and Safe Sets

To explicitly incorporate collision-avoidance requirements at intersections into the planning process, this paper uses a control barrier function (CBF) formulation [26] to model local safety-related feasibility constraints. Unlike approaches that check collisions only after trajectories have already been propagated, the proposed CBF/TTCBF formulation is used as a pre-expansion screening mechanism. In other words, the filter is embedded inside the search pipeline so that locally unsafe actions can be removed before additional tree-search budget is consumed.

#### 2.4.1. Obstacle Function Construction

Given the vehicle’s rectangular shape, performing precise rectangular collision detection for multiple vehicles and multiple candidate actions in online planning incurs significant computational overhead. This paper employs a multi-circle coverage approach to approximate the vehicle geometry. Let the centers of the corresponding sub-circles for the self-vehicle and obstacle vehicle be denoted by $p_{e}^{i}$ and $p_{o}^{j}$, with radii denoted by $r_{e}^{i}$ and $r_{o}^{j}$, respectively. With an additional safety margin of $d_{safe}$, the obstacle function can be defined as:

(5) $$h(x)={∥p_{e}^{i}-p_{o}^{j}∥}^{2}-(r_{e}^{i}+r_{o}^{j}+d_{safe}{)}^{2}$$

where x denotes the combined state formed by the relative geometric relationship between the own vehicle and the obstacle vehicle; $∥\cdot ∥$ represents the Euclidean norm. When $h\left(x\right)>0$, it indicates that a safe distance is maintained between the two vehicles; when $h\left(x\right)=0$, the system is within the safety boundary; when $h\left(x\right)<0$, it signifies the presence of collision risk.

#### 2.4.2. Safe Set and Forward-Invariance Condition

Based on the obstacle function, the system’s safe set is defined as:

(6) C={x∈X:h(x)≥0}

Here, C denotes the safe set, and X denotes the system state space. For the discrete-time system $x_{t+1}=f(x_{t},u_{t})$, if the control input can satisfy:

(7) $$h(x_{t+1})\geq (1-\gamma )h(x_{t})$$

Rather than being interpreted as a global formal safety guarantee, this condition is used as a computationally tractable criterion for local safety-aware action screening under the short-horizon approximation adopted in this paper. The relaxation factor γ adjusts the conservativeness near the safety boundary. Equation (7) therefore serves as the basis for pre-expansion pruning of locally unsafe actions.

### 2.5. Planning Problem Statement

Based on the above modeling, the online planning problem at an unsignalized intersection can be stated as follows: given the current observed state *s*, find a continuous action sequence $U_{t}=\left\{u_{t},u_{t+1},\ldots ,u_{t+H-1}\right\}$ over a finite planning horizon *H* that balances safety, task completion, efficiency, and control smoothness while respecting vehicle dynamics and local feasibility constraints:

(8) $$\underset{U_{t}}{max}\;E\left[\sum_{k=0}^{H-1}\;\gamma_{r}^{k}r_{t+k}\right]$$

**Figure 2:**
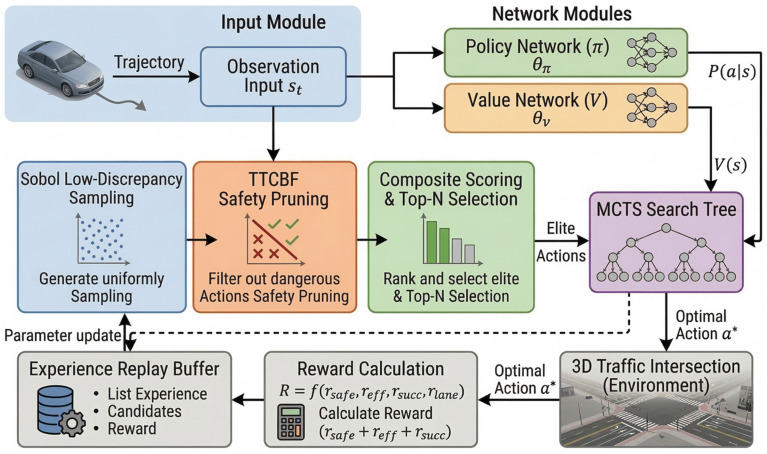
Framework overview of CP-LDS-MCTS. The proposed expansion-stage pipeline coordinates candidate generation, safety pruning, and elite-action selection.

The constraints are:

(9a) $$x_{t+k+1}=f(x_{t+k},u_{t+k}),\;k=0,\ldots ,H-1$$

(9b) $$u_{t+k}\in U,\;k=0,\ldots ,H-1$$

(9c) $$h(x_{t+k+1})\geq (1-\gamma )h(x_{t+k}),\;k=0,\ldots ,H-1$$

where $\gamma_{r}$ represents the reward discount factor, and U denotes the admissible control set. Due to the uncertainty in other vehicles’ behaviors within the environment, the continuous action space, and limited search budget, directly solving Equation (8) presents significant challenges. Therefore, this paper introduces a dual-network-guided Monte Carlo Tree Search (MCTS) as an approximate solver, with a focus on enhancing its candidate action generation and safety screening mechanisms. The algorithmic framework is illustrated in Figure 2.

### 2.6. Policy-Value Dual Network Guidance

#### 2.6.1. Dual-Network Architecture

To reduce the computational overhead of random simulations and enhance search directionality, the method adopts a dual-network architecture where the policy network $\pi_{\theta }$ and value network $V_{\phi }$ operate in tandem. The policy network takes the current state $s_{t}$ as input and outputs the parameters of a two-dimensional Gaussian distribution over the control vector $u_{t}$:

(10) $$N\left(\mu ,\Sigma \right),\;\;\Sigma =diag(\sigma_{a}^{2},\sigma_{\delta }^{2})$$

where μ denotes the mean action vector under the current state, while Σ denotes the covariance matrix. Equivalently, the standard-deviation vector σ used in the Sobol affine mapping below satisfies Σ = diag(σ⊙σ). This output is used both as the prior for PV-MCTS and as the target distribution for the Sobol-based candidate generation proposed in this paper.

The value network takes state $s_{t}$ as input and outputs a scalar value estimate $V_{\phi }(s_{t})$, which replaces expensive random simulation in conventional MCTS rollouts. Both networks use three fully connected hidden layers. This design keeps the model lightweight enough for online inference while still providing informative guidance to the search process.

#### 2.6.2. Training Reward Design

Because the training objective must reflect safety, task completion, efficiency, and control smoothness simultaneously, the instantaneous reward is designed as a weighted combination of three components:

(11) $$r_{t}=w_{safe}\cdot R_{safe}+w_{succ}\cdot R_{succ}+w_{eff}\cdot R_{eff}$$

where the safety incentive is defined as:

(12) $$R_{safe}=\left\{\begin{array}{c} R_{coll},\;if\;collision\\ -w_{d}/(d_{min}+\varepsilon ),\;\;otherwise \end{array}\right.$$

Mission completion rewards provide positive incentives when vehicles successfully reach target zones; efficiency rewards are defined as:

(13) $$R_{eff}=v_{t}/v_{max}-c_{time}$$

In these expressions, $w_{safe}$, $w_{succ}$ and $w_{eff}$ represent the safety, success, and efficiency weights, respectively; the mission-completion reward term in Equation (11) is denoted by $R_{succ}$, and the distance-shaping coefficient inside the safety reward is denoted by $w_{d}$; $R_{coll}$ is the collision penalty constant; $d_{min}$ is the minimum distance between the ego vehicle and surrounding vehicles; ε is a small positive number used to avoid a zero denominator; $v_{max}$ is the maximum speed limit of the scenario; and $c_{time}$ is the time penalty coefficient. The reward design shapes behavior during learning, whereas TTCBF pruning acts during online inference. The two mechanisms therefore play different roles: the former biases the policy away from risky states in training, and the latter enforces a local feasibility filter during planning.

## 3. Methods

### 3.1. Low-Discrepancy Action Sampling Based on Sobol Sequences

#### 3.1.1. Motivations for Low-Discrepancy Sampling

Conventional continuous-action MCTS often draws candidate actions by pseudo-random sampling from the Gaussian prior output by the policy network. When the number of samples is limited, however, pseudo-random draws may produce both local clustering and large uncovered regions. In a budget-constrained online planner, these coverage defects directly reduce the probability that the search will evaluate high-quality feasible actions.

To improve the quality of distribution coverage within a limited sample budget, this paper introduces the Sobol low-discrepancy sequence to generate initial candidate actions [27,28]. The motivation can be explained through the Koksma-Hlawka inequality, which links numerical integration error to sample discrepancy:

(14) $$\left|\int f(x)dx-\frac{1}{N}\sum_{i=1}^{N}f(x_{i})\right|\leq V(f)\cdot D^{*}(x_{1},\ldots ,x_{N})$$

where f denotes the integrand defined on the unit hypercube, $x_{i}$ represents the sample point, N denotes the number of candidate-action samples, V(f) represents the function variation, and $D^{*}$ denotes the star-discrepancy metric of the sample set. Compared to pseudorandom sequences, Sobol sequences provide more balanced spatial coverage with a smaller sample size, making them more suitable for candidate action generation under budget constraints.

#### 3.1.2. Mapping from Sobol Sequences to Action Samples

Suppose the policy network outputs a Gaussian distribution $N\left(\mu ,\Sigma \right)$. The Sobol sequence is mapped to the target action space in the following three steps:

1. Generate a standard Sobol pattern. Generate *N* Sobol sample points $u_{i}$ within a two-dimensional unit hypercube ${\left[0,\;1\right]}^{2}$;
2. Perform an inverse cumulative distribution transformation. Use the inverse cumulative distribution function of the standard normal distribution $\Phi^{-1}\left(\cdot \right)$ to obtain a standard normal sample:

(15) $$z_{i}^{k}=\Phi^{-1}(u_{i}^{k}),k=1,\ldots ,d_{a}$$

3. Map the standard Gaussian samples to the target action distribution by affine transformation, thereby producing candidate continuous actions:

(16) $$u_{i}=\mu +\sigma ⊙z_{i}$$

where $d_{a}$ represents the action dimension, with $d_{a}\;=\;2$ in the present setting; $u_{i}^{k}$ represents the component of the i-th sample in the k-th dimension; $z_{i}^{k}$ represents the corresponding standard normal sample; μ and σ denote the mean vector and standard deviation vector of the policy network’s output, respectively; and ⊙ denotes the Hadamard element-wise product. This yields the set of candidate actions $U_{init}=\{u_{1},\ldots ,u_{N}\}$, where each $u_{i}$ is a sampled two-dimensional control vector. This process preserves the prior distribution of the policy network’s output while utilizing low-discrepancy sequences to improve coverage uniformity under a limited sample budget. For the two-dimensional action space in this paper, the introduction of low-discrepancy sampling does not significantly increase the computational burden, yet it directly improves the quality of the candidate action set, a point that will be further demonstrated through experiments in the following sections.

### 3.2. A Safety Pruning Mechanism Based on TTCBF

#### 3.2.1. Discrete-Time Impulse Response Expansion

For each sampled action vector in the candidate set $U_{init}$, it is necessary to determine whether it still satisfies the forward invariance requirement of the safety set after a single-step expansion. This determination is not based on the static assumption of “unknown background traffic” but rather on the observable states of neighboring vehicles at the current time and their short-term local extrapolations. Direct computation $h(x_{t+1})$ would be affected by nonlinear vehicle dynamics and multi-object interactions, resulting in significant computational overhead. To address this, this paper draws on the discrete-time approximation approach of TTCBF [29] and performs a second-order Taylor expansion of the obstacle function at the current state:

(17) $$h(x_{t+1})\approx h(x_{t})+\nabla h\cdot \Delta x+\frac{1}{2}\Delta x^{T}\nabla^{2}h\cdot \Delta x$$

Let the relative position and relative velocity be $\Delta p=p_{ego}-p_{obs}$ and $\Delta v=v_{ego}-v_{obs}$, respectively; then the first- and second-order terms can be approximated as:

(18a) $$\nabla h\cdot \Delta x=2\Delta p^{T}\Delta v\cdot \Delta t$$

(18b) $$\frac{1}{2}\Delta x^{T}\nabla^{2}h\cdot \Delta x=({∥\Delta v∥}^{2}+\Delta p^{T}\Delta a_{rel})\cdot \Delta t^{2}$$

In single-step extension, the future state of the background vehicle is obtained by extrapolating its current observed values using short-term first-order motion; if the acceleration of the neighboring vehicle is not explicitly estimated, a value of $a_{obs}=0$ from the local prediction is used as a simplified approximation. According to the kinematic bicycle model, the acceleration of the self-vehicle can be expressed as:

(19) $$a_{ego}=a_{lon}\cdot e_{t}+\frac{v^{2}tan\delta_{f}}{L}\cdot e_{n}$$

where $e_{t}$ and $e_{n}$ represent the tangent and normal unit vectors, respectively.

#### 3.2.2. Formulation of Affine Inequality Constraints

By substituting Equation (19) into Equation (17) and combining it with the discrete-time feasibility condition in Equation (7), the local safety requirement can be rewritten as an affine inequality with respect to control input under the TTCBF framework:

(20) $$A\cdot a_{lon}+B\cdot \tan\delta_{f}\geq C$$

Among these:

(21a) $$A=2\Delta p^{T}e_{t}\cdot \Delta t^{2}$$

(21b) $$B=2\Delta p^{T}e_{n}\cdot v^{2}/L\cdot \Delta t^{2}$$

(21c) $$C=(\gamma -1)h(x_{t})-2\Delta p^{T}\Delta v\cdot \Delta t-{∥\Delta v∥}^{2}\cdot \Delta t^{2}$$

Equation (20) therefore provides a fast criterion for screening candidate actions. When multiple surrounding vehicles are present, the feasible action set $U_{safe}$ is obtained by intersecting the corresponding local safety constraints. Compared with performing collision checks only after deeper tree expansion, this pre-expansion pruning strategy reallocates computation toward actions that are more likely to be both safe and useful.

#### 3.2.3. Local Approximation Error and Safety Interpretation

The TTCBF pruning rule used in this paper is derived from a second-order Taylor expansion of the obstacle function over one planning step. Let $x\left(\tau \right)$ denote the local relative-state trajectory over $\tau \in \left[t,t+\Delta t\right]$, and assume that $h\left(x\right)$ is three-times continuously differentiable in a neighborhood of this trajectory. If the relative motion of the ego vehicle and neighboring vehicles remains bounded during one planning step, then the neglected third-order remainder admits the Lagrange-form bound:

(22) $$R_{3}\leq \frac{C_{h}\Delta t^{3}}{6}$$

where $C_{h}$ upper-bounds the third-order directional derivative of h along the local motion trajectory. Hence the local truncation error is $O\left(\Delta t^{3}\right)$. A practically useful consequence is that if the approximate TTCBF inequality is satisfied with an additional slack η chosen not smaller than the local remainder bound, then the exact one-step barrier variation remains conservative up to the first neglected order. In this sense, η acts as a robustness buffer: it converts approximation uncertainty near the boundary into intentional conservativeness rather than leaving it as uncontrolled optimism.

At the same time, this argument is strictly local. It relies on three assumptions: first, the one-step motion remains inside the neighborhood in which the Taylor expansion is valid; second, neighboring vehicles do not execute strongly unmodeled maneuvers within Δt; and third, the short-horizon extrapolation error is smaller than the selected safety buffer. Therefore, the TTCBF rule should be interpreted as a local safety-aware screening criterion, not as a global formal safety guarantee under arbitrary surrounding-agent behavior or long-horizon prediction mismatch.

In implementation, conservativeness is reinforced in two additional ways. First, the screening condition uses the pruning margin η, so candidate actions close to the estimated safety boundary are discarded rather than retained. Second, when the safe candidate set becomes empty, the planner invokes emergency braking instead of forcing expansion from a highly uncertain boundary region. These design choices do not replace formal closed-loop verification, but they reduce the probability that approximation or prediction error propagates into obviously unsafe child nodes while preserving the lightweight character required by online MCTS expansion.

After TTCBF pruning, all actions in the safe candidate set $U_{safe}\;$ satisfy the local one-step feasibility condition, but they may still differ substantially in long-term return, traversal efficiency, and consistency with the policy prior. Randomly choosing a few actions from this safe set would underuse the information already available from the policy and value networks.

For any safe candidate action $u_{i}\in U_{safe}$, its composite score is defined as:

(23) $$Score(u_{i})=w_{P}\cdot Norm(\pi_{\theta }(u_{i}|s))+w_{V}\cdot Norm(V_{\phi }(f(s,u_{i})))$$

In this context, the prior term of the policy is given by the probability density function of a Gaussian distribution evaluated in the same action dimension $d_{a}$ introduced in Equation (16):

(24) $$\pi_{\theta }(u|s)=\frac{1}{\left(2\pi {)}^{d\_a/2}|\Sigma {|}^{1/2}\right.}exp\left(-\frac{1}{2}(u-\mu {)}^{T}\Sigma^{-1}(u-\mu )\right)$$

The normalization function uses the Min-Max form over the current safe candidate set:

(25) $$Norm(x)=\frac{x-x_{min}}{x_{max}-x_{min}}$$

In Equation (23), $w_{P}$ measures the consistency between the action and the policy prior, while $w_{V}$ measures the long-term value associated with the action. Ultimately, the Top-K elite actions with the highest scores are selected to form the elite set $U_{elite}$, which is used for subsequent tree expansion. The purpose of this design is not to simply favor a single score, but to balance “short-term feasibility” and “long-term profitability” while ensuring safety.

### 3.3. Algorithm Planning Process

After integrating the above modules, the complete execution flow of CP-LDS-MCTS is summarized in Algorithm 1 and Figure 2. The selection phase uses a prior-guided UCT rule, the expansion phase applies Sobol sampling, TTCBF safety pruning, and composite scoring, and the evaluation phase uses the value network for fast state assessment. The method is designed so that the root candidate set remains fixed during one planning cycle after pruning and elite selection, which keeps action comparisons internally consistent within a single search call.

The UCT formula used in the selection phase is:

(26) $$UCT(s,u)=Q(s,u)+c_{puct}\cdot P(s,u)\cdot \frac{\sqrt{N(s)}}{1+N(s,u)}$$

The backpropagation phase uses incremental mean updating:

(27) $$Q(s,u)\leftarrow Q(s,u)+\frac{v-Q(s,u)}{N(s,u)}$$

where Q(s,u) represents the cumulative value estimate of the state-control pair (s,u); P(s,u) represents the prior probability provided by the policy network; N(s) represents the number of times the state has been visited; N(s,u) represents the number of times the state-control pair has been visited; $c_{puct}$ represents the exploration coefficient; and v represents the backpropagated value of the current leaf node as evaluated by the value network.
**Algorithm 1** CP-LDS-MCTS Planning Algorithm Pseudocode**input:** Status $s_{t}$, Policy Network $\pi_{\theta }$, Value Network $V_{\phi }$, Number of simulations $N_{sim}$, Maximum depth *D*, Number of candidate actions N, Number of elite actions $K_{elite}$ **output:** Optimal action $u^{*}$ 1: Initialize the root node *root* ← CreateNode($s_{t}$) 2: **for** k = 1 **to** $N_{sim}$ **do** 3:  *node* ← *root* 4:  **while** node is not a leaf node and depth < *D* **do** 5:    u ←$arg\;\underset{u}{max}\;\left[Q(s,u)+c\cdot P(s,u)\cdot \frac{\sqrt{N(s)}}{1+N(s,u)}\right]$ 6:    *node* ← Child(*node*, u) 7:   **end while** 8:    (μ, Σ) ←$\pi_{\theta }$(*node.state*) 9:    $U_{init}$ ← SobolSample(N, μ, Σ) 10:   $U_{safe}$ ← TTCBFPrune($U_{init}$, *node.state*) 11:   **if** $\left|U_{safe}\right|=\emptyset$ **then** 12:    $U_{safe}$ ← EmergencyBrake() 13:   **end if** 14:   $U_{elite}$ ← TopKSelect($U_{safe}$, $\pi_{\theta }$, $V_{\phi }$, $K_{elite}$) 15:   **for** each ${u\in U}_{elite}$ **do** 16:    Create a child node and add it to the search tree 17:   **end for** 18:   v ←$V_{\phi }$(*node.state*) 19:   **while** *node* ≠ *root* **do** 20:      N(s,u) ← N(s,u) + 1 21:      Q(s,u) ← Q(s,u)+(v−Q(s,u))/N(s,u) 22:      *node* ← Parent(*node*) 23:   **end while** 24: **end for** 25: $u^{*}$ ← ${argmax}_{u}\;N(root,u)$ 26: **return** $u^{*}$


### 3.4. Computational Complexity Analysis

The online cost of CP-LDS-MCTS can be decomposed into four parts: tree selection/backpropagation, candidate generation, TTCBF pruning, and elite-action scoring. Let M be the number of simulations, D the maximum depth, N the number of candidate actions, $n_{nb}$ the number of neighboring vehicles considered in safety screening, and K the number of elite actions retained for expansion. Under a fixed-size heap implementation for Top-K selection, the dominant worst-case cost per planning step is $O\left(MD+N+N\cdot n_{nb}+N_{safe}\log K\right)$, where $N_{safe}\leq N$ denotes the number of candidates that remain after pruning. The MD term comes from repeated tree traversal and backup; the linear N term from Sobol candidate generation and Gaussian mapping, the $N\cdot n_{nb}$ term from screening each candidate against nearby vehicles, and the $N_{safe}\log K$ term from maintaining the elite-action heap.

This decomposition clarifies where CP-LDS differs from PV-MCTS and Grid-MCTS. The additional pre-expansion cost appears mainly in the $N\cdot n_{nb}$ screening term and the ranking term, but this extra work also reduces the effective branching burden of the subsequent search. In PV-MCTS and Grid-MCTS, many weak or marginally feasible actions can still consume expansion and evaluation budget. In CP-LDS, only the safe and highest-scoring subset is expanded, so the downstream search operates on a smaller and more informative child set. The auxiliary memory overhead is likewise modest, because the expansion stage stores only the candidate set, the screening results, and a fixed-size Top-K heap, yielding O(N+K) extra memory beyond the tree itself. Real-Time Feasibility and Statistical Analysis Section complements this asymptotic analysis with measured latency statistics, so that the computational claim is supported both analytically and empirically.

## 4. Experiments

### 4.1. Experimental Design and Evaluation Criteria

**Figure 3:**
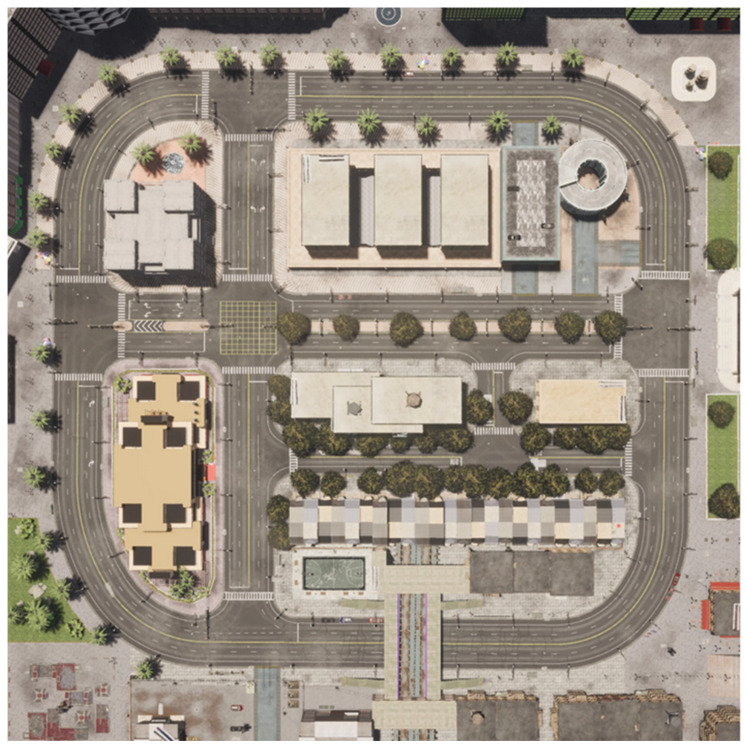
Stylized CARLA evaluation scenario used in Town03.

To validate the effectiveness of the proposed method, this paper conducts simulation experiments in CARLA 0.9.14 [30] using a four-way unsignalized intersection in Town03, as shown in Figure 3, with a central conflict zone of approximately 15 m × 15 m. Three traffic densities are considered according to the average number of surrounding vehicles: low density (3 vehicles), medium density (7 vehicles), and high density (12 vehicles). For each method and each density, 200 evaluation episodes are collected for each of the five random seeds. In addition to PV-MCTS and Grid-MCTS, two stronger baselines are introduced in the revised manuscript: PPO [31], which directly outputs continuous acceleration and steering commands from the same state representation and reward design, and MPC-CBF [21], which combines a short-horizon kinematic MPC objective with a CBF-based safety filter. All methods share the same vehicle model, action bounds, and scenario settings to preserve fairness. Key experimental parameters are listed in Table 1.

The evaluation considers four dimensions: safety, task completion, efficiency, and control smoothness. The corresponding metrics are Safety Rate (SR), Success Rate (SCR), Average Travel Time (ATT), and Path Smoothness (PS). All reported values are presented as mean ± standard deviation across repeated runs. For each method and density, five random seeds are used, with 200 evaluation episodes per seed. The main medium- and high-density comparisons are further examined using two-sided Welch *t*-tests, which were chosen because equal variance across methods was not assumed. To improve reproducibility, the implementation uses a three-circle vehicle approximation with a radius of 0.95 m, a safety margin of 0.30 m, and Poisson background-vehicle arrivals calibrated to produce the three target traffic regimes. Runtime measurements are collected on an Intel Core i7-12700H CPU with 32 GB RAM (Intel Corporation, Santa Clara, CA, USA); online latency is measured with single-step planning executed on the CPU for fair comparison across methods.

### 4.2. Parameter Selection, Sampling Quality, and Training Stability

The main hyperparameters of CP-LDS are the candidate sampling size *N* and the composite-score weight pair $\left(w_{P},w_{V}\right)$. These quantities determine, respectively, the coverage quality of the candidate set and the relative emphasis placed on policy prior versus value estimation during elite-action selection. Their effects are therefore examined jointly in terms of planning performance, convergence behavior, and runtime overhead.

**Figure 4:**
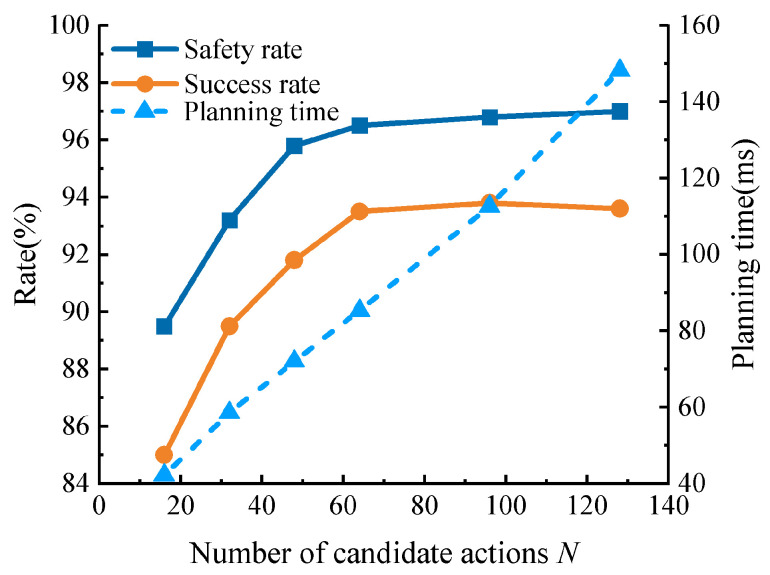
Effect of candidate action sampling size *N* on safety rate, success rate, and planning time in the medium-density scenario.

Figure 4 plots the measured sensitivity of the expansion stage to the candidate-set size *N* in the medium-density scenario. As *N* increases from 16 to 64, the safety rate rises from 89.5% to 96.5% and the success rate rises from 85.0% to 93.5%, while the average planning time increases from 42.3 ms to 85.4 ms. When *N* is further increased to 96 and 128, the performance gain becomes marginal: SR improves only from 96.5% to 96.8% and 97.0%, and SCR changes only from 93.5% to 93.8% and 93.6%, whereas the mean planning time increases to 112.7 ms and 148.3 ms. This pattern indicates a clear diminishing-return regime beyond *N* = 64; therefore, *N* = 64 is adopted as the default setting for the remainder of the study.

**Figure 5:**
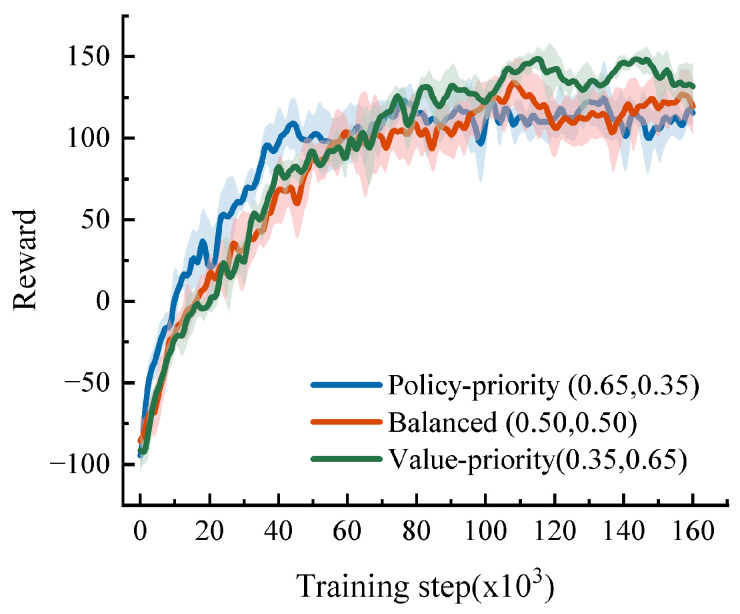
Training convergence curves for different combinations of composite-score weights.

With N fixed at 64, three composite-score settings are further compared: policy-priority (*w_p_* = 0.65, *w_v_* = 0.35), balanced (*w_p_* = 0.50, *w_v_* = 0.50), and value-priority (*w_p_* = 0.35, *w_v_* = 0.65). Figure 5 shows that the policy-priority setting improves faster during the early stage, whereas the value-priority setting overtakes it later and reaches the highest final reward level. At the end of training, the mean reward of the value-priority setting is 131.67, compared with 119.50 for the balanced setting and 115.37 for the policy-priority setting. Table 2 confirms that this difference is not limited to one reward curve. In the medium-density scenario, the value-priority setting reaches 97.5% SR and 94.5% SCR, versus 93.0% and 90.5% for the balanced setting and 92.0% and 89.0% for the policy-priority setting, while also reducing ATT from 17.2–17.5 s to 15.2 s. These results indicate that stronger value emphasis improves long-horizon decision quality rather than merely encouraging aggressive behavior.

**Figure 6:**
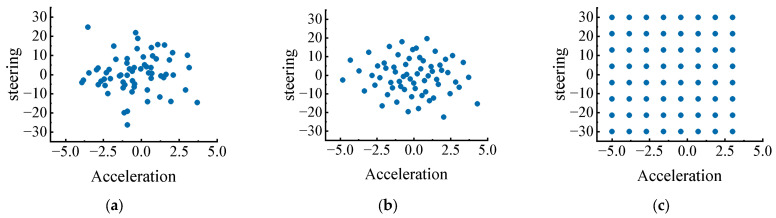
Comparison of candidate-action distributions under the same Gaussian prior: (**a**) pseudo-random sampling in PV-MCTS; (**b**) Sobol low-discrepancy sampling in CP-LDS; (**c**) uniform grid sampling in Grid-MCTS.

**Figure 7:**
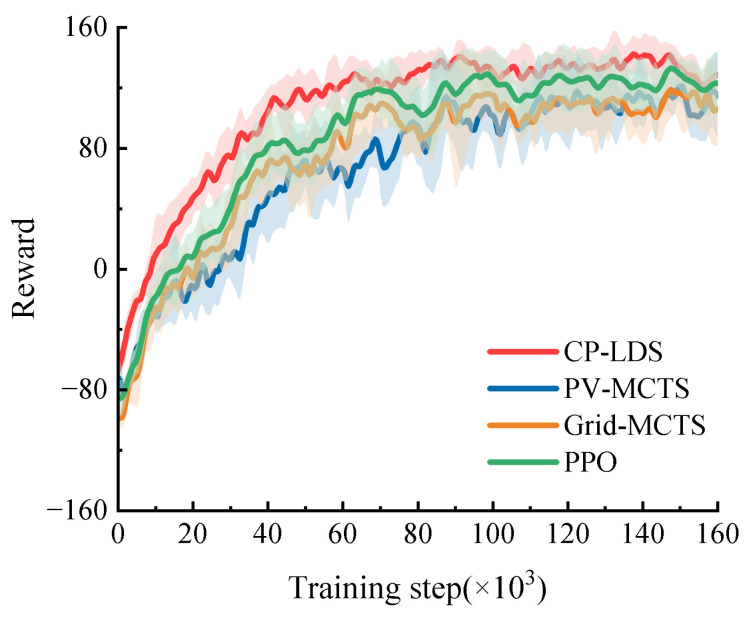
Comparison of training convergence for PV-MCTS, Grid-MCTS, PPO, and CP-LDS.

The effect of candidate-generation strategy is also visible in the sampling layouts and convergence curves. Figure 6 shows that pseudo-random sampling in PV-MCTS exhibits local clustering and uncovered regions, whereas Grid-MCTS provides regular spacing but ignores the shape of the learned prior. In contrast, Sobol sampling preserves the prior structure while distributing samples more evenly over the likely action region. The corresponding training curves in Figure 7 show that CP-LDS converges faster after the early exploration stage and reaches the highest final reward. At 160,000 training steps, the mean return of CP-LDS is 128.59, compared with 123.19 for PPO, 114.83 for PV-MCTS, and 106.09 for Grid-MCTS. Moreover, CP-LDS finishes with smaller reward dispersion than PPO and substantially smaller dispersion than the original search-based baselines, which is consistent with the interpretation that better candidate coverage reduces unstable search behavior during training.

### 4.3. Comparison of Overall Performance Under Different Traffic Densities

**Figure 8:**
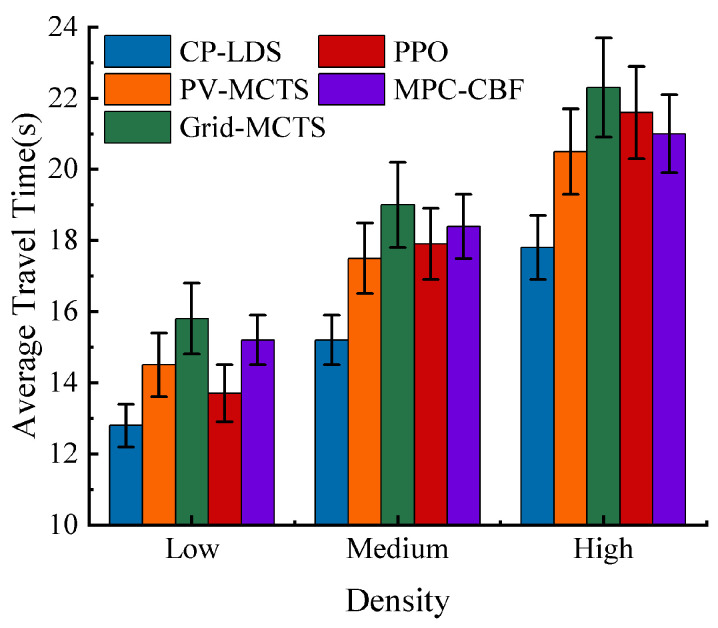
Comparison of average travel times under different traffic densities for PV-MCTS, Grid-MCTS, PPO, MPC-CBF, and CP-LDS.

**Figure 9:**
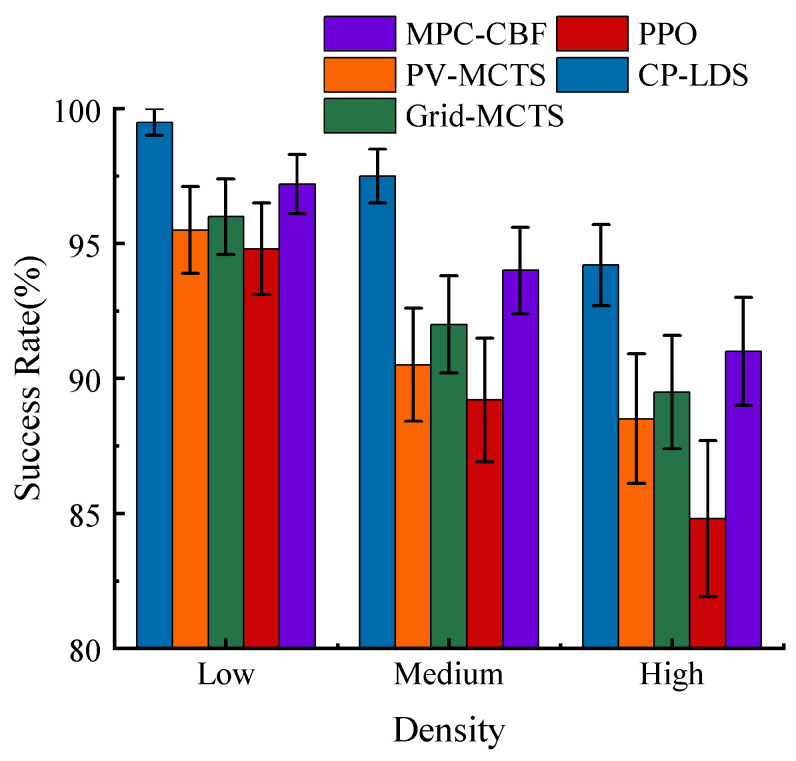
Comparison of success rates under different traffic densities for PV-MCTS, Grid-MCTS, PPO, MPC-CBF, and CP-LDS.

**Figure 10:**
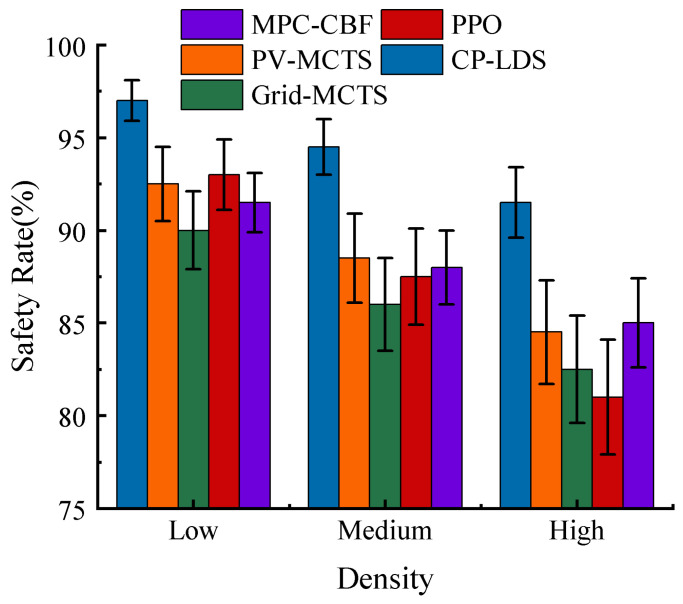
Comparison of safety rates under different traffic densities for PV-MCTS, Grid-MCTS, PPO, MPC-CBF, and CP-LDS.

Under the selected parameter setting, the overall performance of the compared methods under different traffic densities is summarized in Table 3 and Figure 8, Figure 9 and Figure 10. As expected, all methods degrade as traffic density increases, but the degradation rate differs substantially. CP-LDS achieves the best overall balance across the reported metrics in all three densities. In low density, it attains 99.5% SR and 97.0% SCR while also achieving the shortest average travel time of 12.8 s. In medium density, it reaches 97.5% SR and 94.5% SCR, compared with 94.0% and 88.0% for MPC-CBF, 92.0% and 86.0% for Grid-MCTS, 90.5% and 88.5% for PV-MCTS, and 89.2% and 87.5% for PPO. In high density, it maintains 94.2% SR and 91.5% SCR, whereas MPC-CBF drops to 91.0% SR and 85.0% SCR, the original MCTS baselines drop to the 88.5–89.5% SR range and the 82.5–84.5% SCR range, and PPO decreases further to 84.8% SR and 81.0% SCR. Across the five-method comparison, PPO remains competitive in low-density efficiency but deteriorates more quickly as interaction complexity grows, while MPC-CBF is safer than PPO but becomes more conservative in success rate and travel time.

A joint reading of Figure 8, Figure 9 and Figure 10 indicates that the observed improvement is not a simple “safer but slower” trade-off. Grid-MCTS benefits from regular coverage but suffers from lower success rate and longer travel time because the fixed grid does not adapt to the learned action prior. PPO provides competitive travel efficiency in easier scenes, but its direct policy output is more sensitive to interaction complexity and shows weaker safety consistency in denser traffic. MPC-CBF offers a strong safety-oriented baseline, yet its conservative control bias leads to longer average travel time and lower completion efficiency. By contrast, CP-LDS improves safety, success rate, and travel time simultaneously relative to the original MCTS baselines, which suggests that the benefit comes from reallocating search budget toward actions that are both locally feasible and strategically useful.

#### Real-Time Feasibility and Statistical Analysis

Real-time feasibility is critical for an online driving planner. Table 4 reports the measured single-step planning latency on the evaluation platform under CPU execution. As expected, PPO is the fastest because it requires only one policy forward pass, whereas MPC-CBF is the slowest because it solves an optimization problem at each step. CP-LDS introduces additional pre-expansion computation relative to PV-MCTS, but its mean latency remains 24.7 ms and its 95th-percentile latency remains 31.2 ms, both far below the 0.5 s planning interval adopted in this work. This means that the added Sobol generation, TTCBF screening, and elite-action selection remain compatible with online execution under the present scenario scale.

Table 5 reports the statistical tests for the main medium- and high-density comparisons. We use two-sided Welch *t*-tests because the variance across repeated runs is not assumed to be equal across methods, and we additionally report Cohen’s d to indicate effect size. Across the tested comparisons, the *p*-values remain below 0.05 and the effect sizes are mostly in the medium-to-large range, which supports the claim that the observed gains are statistically meaningful within the present benchmark setting. At the same time, these tests should be interpreted as within-benchmark evidence rather than universal proof of superiority, because they are conditioned on the current simulator, scenario family, and hyperparameter tuning.

### 4.4. Ablation Experiment

**Figure 11:**
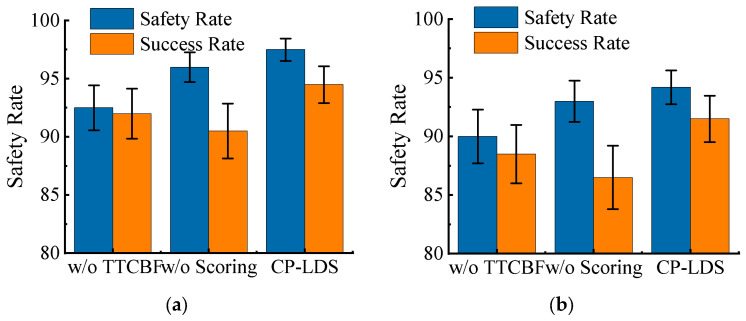
Contributions of each module to safety rate and success rate in medium- and high-density scenarios: (**a**) medium-density scenario; (**b**) high-density scenario.

To further clarify the role of each module in the proposed method, three ablation configurations are considered: (A) CP-LDS w/o TTCBF, which removes the TTCBF-based safety pruning module; (B) CP-LDS w/o Scoring, which retains safety pruning but replaces the composite scoring mechanism with random selection among the Top-K safe actions; and (C) Full CP-LDS, which includes all proposed components. The quantitative results are reported in Table 6, and the corresponding visual comparison is presented in Figure 11.

The ablation results show that the two modules play different but complementary roles in the planning framework. In the medium-density scenario, removing TTCBF pruning reduces SR from 97.5% to 92.5% and SCR from 94.5% to 92.0%, whereas removing composite scoring keeps SR relatively high at 96.0% but lowers SCR to 90.5%. In the high-density scenario, removing TTCBF lowers SR from 94.2% to 90.0%, while removing scoring lowers SCR more sharply from 91.5% to 86.5%. This pattern indicates that TTCBF pruning primarily strengthens the safety baseline by preventing clearly unsafe actions from entering the tree, whereas composite scoring mainly improves the conversion from local feasibility to successful task completion. Taken together, the gain of full CP-LDS does not come from a single isolated module; it arises from the coordinated effect of candidate coverage, local safety filtering, and value-aware expansion priority.

When the composite scoring mechanism is removed, the safety rate remains relatively high, but the success rate declines more clearly than in the full model. This suggests that being safe and feasible is not sufficient for high-quality expansion. Even within the safe action set, it is still necessary to distinguish actions according to their long-horizon value and consistency with the learned policy prior. In this sense, TTCBF pruning provides the safety baseline, whereas composite scoring improves the transition from safety to efficiency.

Taken together, the ablation results confirm that the performance gain of CP-LDS does not come from a single isolated component. Instead, it arises from the coordinated effect of candidate-action coverage, local safety filtering, and value-aware expansion priority. TTCBF pruning prevents search resources from being wasted on unsafe actions, while composite scoring helps the planner focus on actions that are not only feasible but also strategically beneficial. The full CP-LDS therefore achieves the best overall balance between safety and task completion under medium- and high-density traffic conditions.

## 5. Discussion

### 5.1. Explanation of Performance Improvement Mechanisms

As shown in the experimental results, the advantage of CP-LDS does not come from any single isolated module. Instead, it arises from the sequential design of the expansion stage. Sobol low-discrepancy sampling improves the representativeness of the candidate set under a fixed sampling budget; TTCBF pruning removes locally infeasible actions before search effort is wasted on them; and composite scoring concentrates expansion on safe actions with both strong prior plausibility and promising long-horizon value.

The baseline comparisons reinforce this interpretation. Grid-MCTS shows that improving spatial regularity alone is not sufficient when candidate actions are disconnected from the learned action prior. PV-MCTS shows that a learned prior alone is also insufficient when pseudo-random sampling leaves local coverage gaps. The superior performance of CP-LDS therefore reflects the coupling of three properties: prior-consistent candidate generation, local safety-aware filtering, and value-based allocation of search budget.

### 5.2. Implications for the Planning of Intersections Without Traffic Lights

One practical implication of these results is that, for highly interactive scenarios such as unsignalized intersections, safety, success rate, efficiency, and smoothness should not be viewed as fully independent objectives. In online planning, these metrics are linked through the quality of the candidate actions evaluated during search. A planner that finds actions that are simultaneously feasible and strategically useful can improve several metrics at once instead of trading one metric off against another by design.

From an engineering perspective, the proposed method suggests that improving candidate quality can be a more effective use of online computation than simply increasing search depth or the number of rollouts. When the planning budget is tight, the key question is not only how many nodes the search can evaluate, but whether those nodes correspond to actions worth evaluating in the first place. This insight is relevant beyond intersections and may apply to other short-horizon interactive driving tasks with continuous control.

### 5.3. Methodological Limitations and Future Research

Although the method achieves stable results in simulation, several limitations remain. First, the advantage of Sobol low-discrepancy sampling is most evident in low- and medium-dimensional action spaces; the relative benefit may decrease if the action parameterization becomes much higher dimensional. Second, TTCBF pruning is based on short-horizon local prediction and a truncated approximation, so it should be interpreted as a practical local feasibility filter rather than a complete global safety guarantee under arbitrary surrounding-agent behavior. Third, the current validation is still limited to CARLA Town03 and one four-way unsignalized-intersection geometry, and uncontrolled real-world testing has not been conducted at this stage. The contribution of this paper is positioned at the algorithmic level, namely, the coordinated expansion-stage design that couples Sobol low-discrepancy sampling, TTCBF-based safety pruning, and policy-value composite scoring, rather than at the level of on-vehicle deployment. A credible uncontrolled real-world evaluation at an unsignalized intersection would additionally require a complete sensing and localization stack, calibrated actuator dynamics, access to an instrumented test vehicle, and regulatory clearance for interaction with unstructured human-driven traffic. These engineering and safety requirements are substantially beyond the scope of the present algorithmic study. For this reason, the empirical evidence in this work is focused on algorithm-level validation through stronger baselines, multi-density simulation experiments across five random seeds with 200 episodes per seed, two-sided Welch *t*-tests with effect-size reporting, and measured single-step planning latency. In addition, the TTCBF pruning rule is derived under short-horizon assumptions on perception quality and neighbor-vehicle prediction; transferring it to uncontrolled real-world scenarios without first characterizing sensor noise, occlusion, and short-horizon prediction mismatch could overstate the safety claim rather than validate it. Uncontrolled real-world testing, broader intersection geometries, and sensor-level perturbation analysis are therefore identified as important directions for future work. Accordingly, the empirical conclusions of this paper should be interpreted as within-simulator evidence for the proposed expansion-stage coordination, rather than as claims of validated real-world deployment.

## 6. Conclusions

This paper addresses the problem of balancing search efficiency and safety constraints in continuous-action planning at unsignalized intersections by proposing CP-LDS-MCTS, which integrates Sobol low-discrepancy sampling, TTCBF-based safety pruning, and policy-value composite scoring within the expansion stage of MCTS. In the revised manuscript, we clarify that the contribution is not the isolated use of these ingredients, but their coordinated use to improve candidate coverage, reject locally unsafe actions before expansion, and prioritize strategically valuable safe actions under a limited online budget. Within the present CARLA-based evaluation setting, the revised experiments show that this design yields the best overall trade-off among safety, success rate, travel efficiency, and smoothness while remaining compatible with the tested real-time planning interval. The safety property supported by the present results is local and practical rather than a formal global guarantee, because TTCBF-based pruning operates as a one-step approximation-based feasibility filter within the expansion stage and is validated in a simulation-based setting. Formal safety certification and uncontrolled real-world evaluation are left for future work.

## Figures and Tables

**Table 1:** Key Experimental Parameter settings.

Category	Parameters	Value
Vehicle	Wheelbase *L*	2.9 m
	Acceleration range $\left[a_{min},a_{max}\right]$	[−5, 3] m/s^2^
	Steering angle range $\left[\delta_{min},\delta_{max}\right]$	[−30°, 30°]
	Time step ∆t	0.5 s
CP-LDS	Number of samples N	64
	Circle radius r	0.95 m
	Safety margin γ	0.3
	Policy weighting $w_{P}$	0.35
	Value weighting $w_{V}$	0.65
MCTS	Number of simulations $N_{sim}$	10
	Number of elite actions $K_{elite}$	5
	Maximum depth *D*	5
	Exploration constant $c_{puct}$	2.5
Reward	Safety weight $w_{safe}$	1.0
	Success reward $w_{succ}$	2.0
	Efficiency weight $w_{eff}$	0.5
	Collision penalty $R_{coll}$	−100
	Discount factor $\gamma_{r}$	0.99

**Table 2:** Performance of different composite-score weighting combinations under three traffic density conditions.

(*w_p_*, *w_v_*)	Density	SR (%)	SCR (%)	ATT (s)	PS
(0.65, 0.35)	Low	95.5 ± 1.42	92.5 ± 1.86	14.5 ± 0.78	0.082 ± 0.0049
	Medium	92.0 ± 1.97	89.0 ± 2.31	17.2 ± 0.93	0.091 ± 0.0063
	High	89.5 ± 2.26	85.5 ± 2.74	20.1 ± 1.11	0.105 ± 0.0077
(0.50, 0.50)	Low	96.0 ± 1.31	93.0 ± 1.64	14.8 ± 0.72	0.079 ± 0.0044
	Medium	93.0 ± 1.76	90.5 ± 2.08	17.5 ± 0.88	0.088 ± 0.0058
	High	90.5 ± 2.08	86.5 ± 2.43	20.5 ± 1.04	0.098 ± 0.0069
(0.35, 0.65)	Low	99.5 ± 0.58	97.0 ± 1.02	12.8 ± 0.54	0.075 ± 0.0038
	Medium	97.5 ± 0.96	94.5 ± 1.34	15.2 ± 0.63	0.083 ± 0.0049
	High	94.2 ± 1.37	91.5 ± 1.78	17.8 ± 0.81	0.092 ± 0.0057

**Table 3:** Overall performance of PV-MCTS, Grid-MCTS, PPO, MPC-CBF, and CP-LDS under different traffic densities (mean ± standard deviation).

Algorithm	Density	SR (%)	SCR (%)	ATT (s)	PS
PV-MCTS	Low	95.5 ± 1.2	92.5 ± 1.6	14.5 ± 0.6	0.082 ± 0.004
	Medium	90.5 ± 1.7	88.5 ± 1.8	17.5 ± 0.7	0.132 ± 0.006
	High	88.5 ± 1.9	84.5 ± 2.0	20.5 ± 0.9	0.156 ± 0.008
Grid-MCTS	Low	96.0 ± 1.0	90.0 ± 2.0	15.8 ± 0.7	0.145 ± 0.006
	Medium	92.0 ± 1.4	86.0 ± 2.2	19.0 ± 0.8	0.168 ± 0.007
	High	89.5 ± 1.6	82.5 ± 2.4	22.3 ± 1.0	0.195 ± 0.009
PPO	Low	94.7 ± 1.5	91.0 ± 1.8	15.1 ± 0.7	0.118 ± 0.005
	Medium	91.5 ± 1.5	89.0 ± 1.7	17.8 ± 0.8	0.123 ± 0.005
	High	89.8 ± 1.8	85.5 ± 2.0	20.1 ± 0.9	0.139 ± 0.006
MPC-CBF	Low	98.0 ± 0.8	93.5 ± 1.3	14.2 ± 0.5	0.089 ± 0.004
	Medium	95.8 ± 1.1	91.8 ± 1.5	16.4 ± 0.6	0.097 ± 0.004
	High	92.0 ± 1.4	88.4 ± 1.8	19.1 ± 0.8	0.112 ± 0.005
CP-LDS	Low	99.5 ± 0.4	97.0 ± 0.8	12.8 ± 0.5	0.075 ± 0.003
	Medium	97.5 ± 0.6	94.5 ± 1.0	15.2 ± 0.5	0.083 ± 0.003
	High	94.2 ± 0.9	91.5 ± 1.2	17.8 ± 0.6	0.092 ± 0.004

**Table 4:** Planning latency and complexity comparison of the evaluated methods.

Algorithm	Mean Latency (ms)	P95 Latency (ms)	Nominal Candidates	Complexity
PPO	4.1	5.3	1	O (1)
Grid-MCTS	18.9	24.1	64	O (*M* × *D* + *N*)
PV-MCTS	22.4	28.7	64	O (*M* × *D* + *N*)
CP-LDS	24.7	31.2	64	O (*M* × *D* + *N* + *N* × *n_nb_*)
MPC-CBF	31.8	40.5	solver-dependent	O ($n_{v}^{3}$)

**Table 5:** Statistical comparison of CP-LDS against the main baselines in medium- and high-density scenarios.

Density	Metric	Baseline	Welch *t*-Test *p*-Value	Cohen’s d
Medium	SR	PV-MCTS	0.0018	2.03
		Grid-MCTS	0.0004	1.74
		PPO	0.0032	1.65
		MPC-CBF	0.0181	1.09
	SCR	PV-MCTS	0.0025	1.88
		Grid-MCTS	0.0007	2.11
		PPO	0.0048	1.59
		MPC-CBF	0.026	0.96
High	SR	PV-MCTS	0.0034	1.46
		Grid-MCTS	0.0011	1.29
		PPO	0.0075	1.14
		MPC-CBF	0.0228	0.88
	SCR	PV-MCTS	0.0029	1.53
		Grid-MCTS	0.0006	1.85
		PPO	0.0062	1.20
		MPC-CBF	0.0197	0.91

**Table 6:** Ablation results under medium- and high-density traffic conditions (mean ± standard deviation).

Configuration	Density	SR (%)	SCR (%)
CP-LDS w/o TTCBF	Medium	92.5 ± 1.2	92.0 ± 1.4
	High	90.0 ± 1.5	88.5 ± 1.7
CP-LDS w/o Scoring	Medium	96.0 ± 0.9	90.5 ± 1.4
	High	93.0 ± 1.2	86.5 ± 1.8
Full CP-LDS	Medium	97.5 ± 0.6	94.5 ± 1.0
	High	94.2 ± 0.9	91.5 ± 1.2

## Data Availability

The raw data supporting the conclusions of this article will be made available by the authors on request.
